# A Novel Technique to Reduce Anterior Shoulder Dislocation Without Anesthesia - A Prospective Analysis

**DOI:** 10.7759/cureus.33497

**Published:** 2023-01-08

**Authors:** Jayanta K Laik, Ravi Kaushal, Manoj Rajak, Vivek David, Ritesh Kumar, Somit Sarkar

**Affiliations:** 1 Department of Joint Replacement and Orthopedics, Tata Main Hospital, Jamshedpur, IND; 2 Orthopedics, Manipal Tata Medical College, Jamshedpur, IND

**Keywords:** kocher's technique, milch technique, prakash's technique, shoulder reduction, shoulder dislocation

## Abstract

Background

Shoulder dislocation is one of the most common injuries encountered in daily practice. Among shoulder dislocations, anterior shoulder dislocation is the most common variant. There are many techniques described to reduce a dislocated shoulder. Reduction of a dislocated shoulder joint may be performed under anesthesia or without anesthesia. In this study, we have evaluated the efficacy of Prakash's method in reducing anterior shoulder dislocations without anesthesia.

Methodology

This study is a prospective study that was conducted from March 2018 to February 2020 in the department of joint replacement and orthopedics in Tata Main Hospital, Jamshedpur. One hundred two shoulders fulfilling the inclusion criteria were included in this study, and observations were noted.

Results

The results were statistically analyzed, and it was found that this new method was successful in reducing 97.06% shoulders without any anesthesia. Out of the total 102 patients enrolled in this study, 17 (n=102, 16.67%) patients had left-sided shoulder dislocation, and 85 (n=102, 83.33%) patients had right-sided shoulder dislocation. In 91.18% (n=90 out of 102) of the patients, the reduction could be achieved on the first attempt. A second attempt was needed in 7.84% (n=8 out of 102). No complications were noted.

Conclusion

Prakash's method to reduce anterior shoulder dislocation is a simple technique to reduce dislocated shoulders. Through our study, we conclude that it is also an effective technique for reducing anteriorly dislocated shoulders. As there is no requirement of anesthesia, we recommend that orthopedic surgeons as well as emergency care providers should acquaint themselves with this technique.

## Introduction

Shoulder joint dislocation is one of the most common joint dislocations encountered in the casualty department. Mobility at the shoulder joint is gained at the cost of stability. Dislocation of the shoulder joint occurs in 1 to 2% of the population. Its incidence is 1.7% among adults, and it is three times more common among men [1,2,3]. The shoulder can dislocate anteriorly, posteriorly, or inferiorly, and completely or partially, depending upon the mechanism of injury. Anterior shoulder dislocation is the most common type of shoulder dislocation [3,4].

Once the diagnosis is confirmed, various methods are currently described to reduce the dislocated shoulder. The most common method used is Kocher's method. The reduction technique generally depends on the surgeon performing the reduction. Whatever method is used, approximately 70-90% of anterior shoulder dislocations can be reduced by manipulation or closed reduction [5,6,7].

A literature search reveals many methods available for reducing a dislocated shoulder, but every method of reduction is associated with one or more complications. Complications of various methods include neurovascular injuries mainly attributed to traction counter traction method, upper limb dysfunction, and amputation [8].

The literature search suggests a new method of shoulder reduction, namely Prakash's method [9], popularized by an Indian orthopedic surgeon, Dr. L. Prakash. In our study, we have tried to evaluate the efficacy of Prakash's method of shoulder reduction.

## Materials and methods

This study is a prospective study that was conducted from March 2018 to February 2020 in the department of joint replacement and orthopedics in Tata Main Hospital, Jamshedpur. Patients presenting with anterior dislocation of the shoulder and willing to participate in the study were included. Patients with recurrent shoulder dislocation, fracture dislocation, associated neurovascular compromise, associated pelvic and spine injury, associated head injury, and more than two-day-old injuries were excluded from the study.

A complete clinical assessment of the injury was done, and anteroposterior (AP) radiographs of the injured shoulder were taken. The dislocated shoulder was reduced with Prakash's method [9]. Post-reduction check radiographs in the form of AP and lateral views of the shoulder were obtained, and the injured arm was immobilized in a universal shoulder immobilizer.

Data collected was analyzed with appropriate statistical tools, like SPSS version 24 (IBM Inc. Armonk, New York). Student's unpaired T-test was used as the statistical tool to test for the significance of observed mean differences. Statistical analysis was done using the Chi-squared test.

Technique

Reduction is attempted in a sitting position. The patient is made to sit on a chair or bed with their back straight. This is done to fix the scapula. This maneuver can also be attempted with the patient standing, provided the patient is comfortable [9].

First, the patient is counseled and often demonstrated the steps on their opposite shoulder or on a different subject. This is done to gain their confidence. Next, the surgeon holds the dislocated upper limb at the elbow and wrist. 

**Figure 1:**
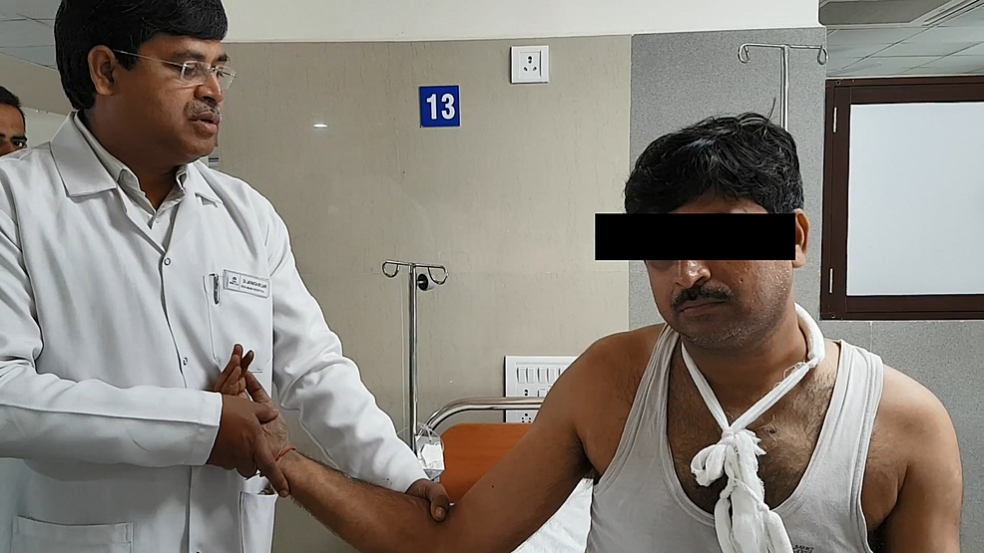
Injured limb is fully externally rotated, keeping the patient comfortable

Now the shoulder is gently externally rotated in the position of deformity to bring the arm into the coronal plane. Maximum external rotation is achieved. Care should be taken to avoid adduction or abduction of the shoulder. The shoulder is maintained in external rotation for over a minute (Figure 1).

**Figure 2:**
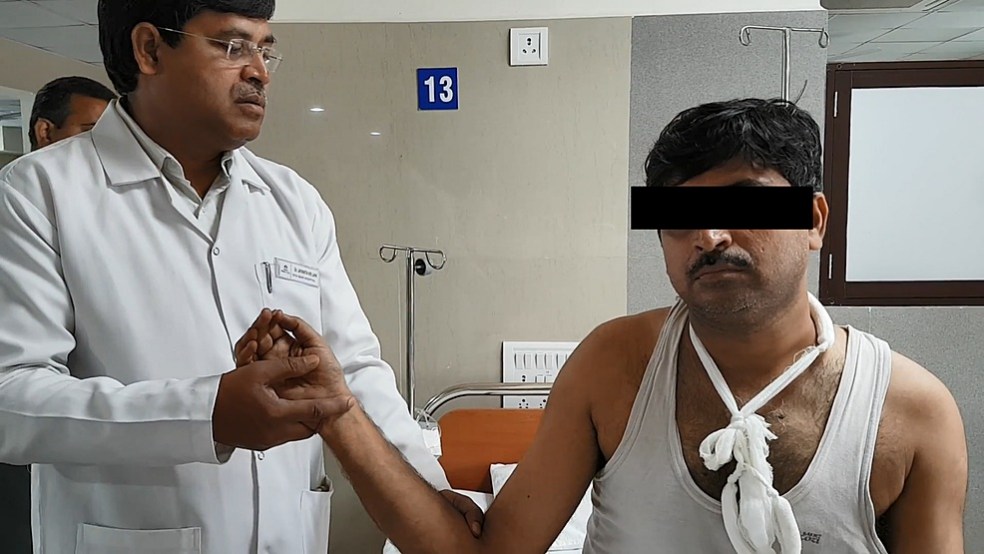
Injured limb is gradually adducted

Next step is crucial as it is the most painful step. Limb has to be maintained in external rotation for around two to three minutes. Try to engage the patient in a conversation so as to distract them. The arm is then gradually adducted in the position of external rotation to bring the elbow over the body (Figure 2).

**Figure 3:**
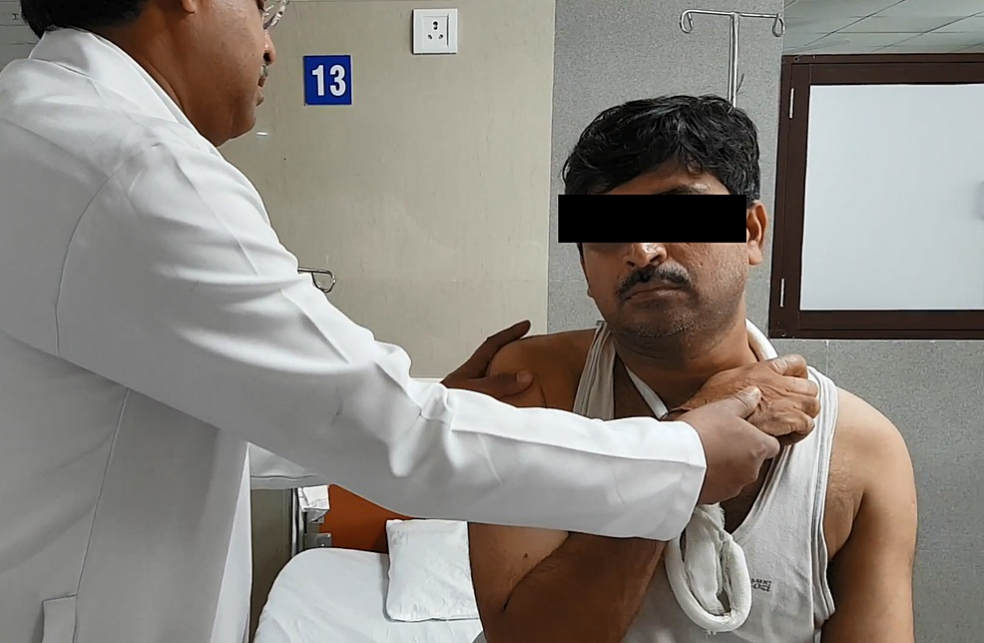
Injured limb is internally rotated to achieve reduction of shoulder

The arm is then gently internally rotated so that the hand touches the opposite shoulder (Figure 3).

The shoulder reduces with a glide without any clunk or audible clicks.

## Results

Out of the total 102 patients enrolled in this study, 79 (n=102, 77.45%) were male, and 23 (n=102, 22.55%) were female. Twenty-three females had a mean age of 41.26 ± 11.70 years, and 79 males had a mean age of 38.32 ± 14.27 years. The overall mean age of the patients was 38.98 ± 13.73 years.

**Figure 4:**
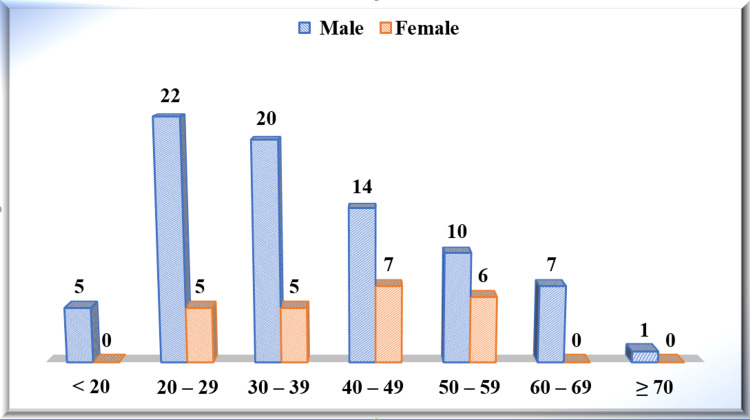
Age and gender distribution among the study participants

The youngest patient to be included in the study was 13 years old; the eldest patient included was 72 years old. Five (4.90%) patients were below 20 years, 27 (26.47%) patients were in the age group of 20 to 29 years, 25 (24.51%) patients were in the age group of 30 to 39 years, 21 (20.59%) patients were in the age group of 40 to 49 years, 16 (15.69%) patients were in the age group of 50 to 59 years, seven (6.86%) patients were in the age group of 60 to 69 years, and one (0.98%) patient was of more than 70 years of age. Here an unparried t-test was used as a test of significance. There were statistically no significant differences among the patients according to their age with gender, with p-value=0.3688 (p>0.05) (Figure 4).

**Figure 5:**
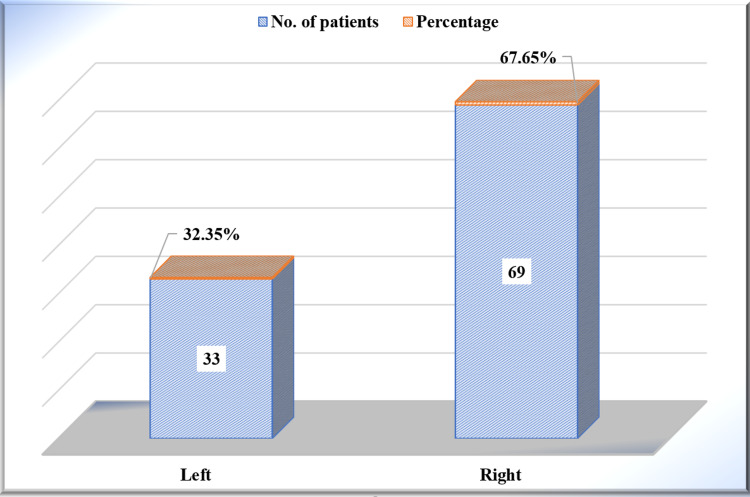
Injured side involved among the study participants

Out of the total 102 patients enrolled in this study, 17 (n=102, 16.67%) patients had left-sided shoulder dislocation, and 85 (n=102, 83.33%) patients had right-sided shoulder dislocation. There was a statistically significant difference with respect to the side of injury, among the patients, with a p-value of <0.0001 using the Chi-squared proportion test (χ2-test) (Figure 5).

**Figure 6:**
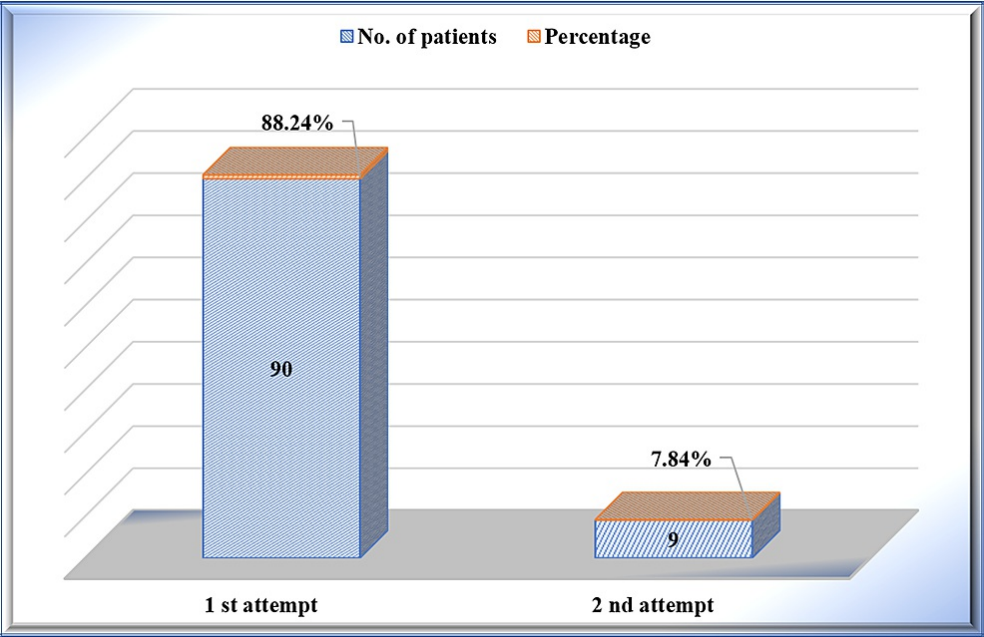
Number of attempts needed to reduce the dislocated shoulder

In 91.18% (n=90 out of 102) of the patients, reduction could be achieved on the first attempt. A second attempt was needed in 7.84% (n=8 out of 102). There was a statistically significant difference with p-value (p<0.000) (Figure 6).

No complication was noted in any of the patients. In the remaining three patients, a reduction could not be achieved even after second attempt, and the procedure had to be abandoned. These shoulders were then reduced under anesthesia using either modified Kocher's or Milch's technique.

**Figure 7:**
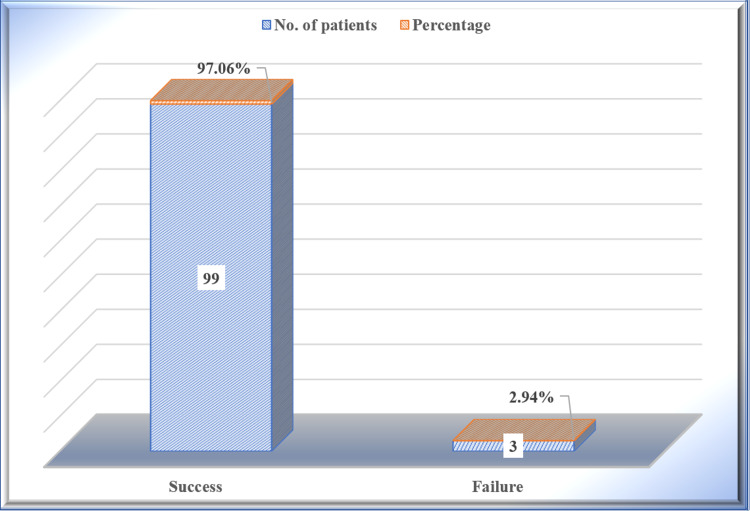
Overall success rate in reducing dislocated shoulders among study participants

To summarize, Prakash's method of shoulder reduction was successful in 97.06% (n=99 out of 102) of patients and failed in 2.94% (n=3 out of 102) patients (Figure 7).

## Discussion

The glenohumeral joint is a ball and socket type of synovial joint between the humerus and scapula. The round head of the humerus articulates with shallow and small glenoid fossa. At any given joint position, only a quarter of the humeral head is in contact with the glenoid cavity. Hence shoulder joint gains mobility at the cost of stability [10].

The range of dislocation varies from 24 to 56 per 100,000 persons per year in different studies. Among various types of shoulder dislocation, anterior shoulder dislocation is the most common type of shoulder dislocation [11,12].

There are various documented techniques in literature for shoulder reduction dating back to 460 BC [13]. Broadly the techniques can be classified into leverage and traction methods. Popular leveraging techniques include Kocher's and Milch's, while popular traction methods are Hippocrates', Stimson's, and the Scapular manipulation technique [14].

Kocher's technique has been known since 1870 [15]. Kocher's technique is associated with a high risk of complications like humeral shaft fracture, axillary vein rupture, rotator cuff injury, and pectoralis major rupture [5,16-18]. Milch's technique was first described by Cooper in 1825 but popularized by Milch in 1938 [19,20]. There is no statistical difference between the two methods, but Milch's method is usually recommended because of fewer complications [20,21].

Traction used in traction methods helps to overcome muscle spasms, and it also facilitates the process of reduction. Hippocrates' method, which uses traction to achieve reduction, is associated with complications like brachial plexus injury and axillary vessel injury [5]. Stimson's technique was described in 1900 and requires the application of weight to the injured limb [22]. Scapular manipulation technique has a high success rate and no complications [23].

An ideal technique for shoulder reduction would be the one that requires the least amount of assistance, can be done with the application of the least amount of force, can be done without sedation, is comfortable for the patient, and is associated with no complications [14].

Prakash's method of shoulder reduction is one such technique that can be performed without sedation and does not require any assistance. It is easy to learn with a shallow learning curve. Reduction can be achieved in the first attempt in almost all of the cases.

In this study, we could reduce 91.18% shoulders in the first attempt, with an overall success rate of 97.06%. There was no need for any assistance. Patients were mostly comfortable throughout the procedure and did not need any analgesia. Due to the apprehensiveness of the patients, the reduction could not be achieved in three patients. However, this study is limited by a small sample size, and we could not compare this method with other traditional methods of shoulder reduction.

## Conclusions

With this study, we conclude that Prakash's method of shoulder reduction is an effective and convenient method of shoulder reduction. This can be performed without the need for any assistance or anesthesia. Orthopedic surgeons must command this technique and put this to use in daily practice. Further comparative studies should be done to compare the effectiveness of this method with other methods of shoulder reduction.

## References

[1] Kazár B, Relovszky E (1969). Prognosis of primary dislocation of the shoulder. Acta Orthop Scand.

[2] Hovelius L (1982). Incidence of shoulder dislocation in Sweden. Clin Orthop Relat Res.

[3] Carrazzone OL, Tamaoki MJ, Ambra LF, Neto NA, Matsumoto MH, Belloti JC (2011). Prevalence of lesions associated with traumatic recurrent shoulder dislocation. Rev Bras Ortop.

[4] Abrams R, Akbarnia H (2021). Shoulder dislocations overview. http://www.ncbi.nlm.nih.gov/books/NBK459125/.

[5] Riebel GD, McCabe JB (1991). Anterior shoulder dislocation: a review of reduction techniques. Am J Emerg Med.

[6] Manes HR (1980). A new method of shoulder reduction in the elderly. Clin Orthop Relat Res.

[7] Anjum R, Pathak S, Sharma AR, Aggarwal J, Sharma A, Pruthi V, Chaudhary AK (2019). Reducing shoulder dislocation without anaesthesia or assistant: validation of a new reduction manoeuvre. Chin J Traumatol.

[8] Waldron VD, Hazel D (1991). Tips of the trade #37. Technique for reduction of shoulder dislocation. Orthop Rev.

[9] Prakash L (2018). A new method for reduction of shoulder dislocations. Ortho Surg Ortho Care Int J.

[10] Lahti A, Andernord D, Karlsson J, Samuelsson K (2016). Shoulder dislocation (Website in Swedish). Lakartidningen.

[11] Dai F, Xiang M, Yang JS, Chen H, Hu XC, Zhang Q, Li YP (2020). Injury mechanism of acute anterior shoulder dislocation associated with glenoid and greater tuberosity fractures: a study based on fracture morphology. Orthop Surg.

[12] Zacchilli MA, Owens BD (2010). Epidemiology of shoulder dislocations presenting to emergency departments in the United States. J Bone Joint Surg Am.

[13] Hussein MK (1968). Kocher's method is 3000 years old. J Bone Joint Surg Br.

[14] Dala-Ali B, Penna M, McConnell J, Vanhegan I, Cobiella C (2014). Management of acute anterior shoulder dislocation. Br J Sports Med.

[15] Theivendran K, Thakrar RR, Deshmukh SC, Dwan K (2019). Closed reduction methods for acute anterior shoulder dislocation. Cochrane Database Syst Rev.

[16] Beattie TF, Steedman DJ, McGowan A, Robertson CE (1986). A comparison of the Milch and Kocher techniques for acute anterior dislocation of the shoulder. Injury.

[17] KI JR (1952). Dislocation of the shoulder complicated by rupture of the axillary vessels. J Bone Joint Surg Br.

[18] Pimpalnerkar A, Datta A, Longhino D, Mohtadi N (2004). An unusual complication of Kocher's manoeuvre. BMJ.

[19] Milch H (1938). Treatment of dislocation of the shoulder. Surg.

[20] Ashton HR, Hassan Z (2006). Best evidence topic report. Kocher's or Milch's technique for reduction of anterior shoulder dislocations. Emerg Med J.

[21] Mattick A, Wyatt JP (2000). From Hippocrates to the Eskimo - a history of techniques used to reduce anterior dislocation of the shoulder. J R Coll Surg Edinb.

[22] Bosley R, Miles J (1979). Scapular manipulation for reduction of anterior inferior dislocations. A new procedure. Presented at the American Association of Orthopedics Surgeons.

[23] Sayegh FE, Kenanidis EI, Papavasiliou KA, Potoupnis ME, Kirkos JM, Kapetanos GA (2009). Reduction of acute anterior dislocations: a prospective randomized study comparing a new technique with the Hippocratic and Kocher methods. J Bone Joint Surg Am.

